# Identification of the cassava *NADP-ME* gene family and its response and regulation in photosynthesis

**DOI:** 10.3389/fpls.2025.1525193

**Published:** 2025-02-27

**Authors:** Haozheng Li, Jin Xiao, Jiahui Chen, Xu Shen, Jia Luo, Fengguang Guo, Shangfei Wang, Liangye Xu, Xin Guo, Shujuan Wang, Haiyan Wang, Wenquan Wang

**Affiliations:** ^1^ School of Tropical Agriculture and Forestry, Hainan University, Danzhou, China; ^2^ College of Agriculture, Guangxi University, Nanning, China; ^3^ National Key Laboratory for Tropical Crop Breeding, Key Laboratory of Biology and Genetic Resources of Tropical Crops, Institute of Tropical Bioscience and Biotechnology, Sanya Research Institute of Chinese Academy of Tropical Agricultural Sciences, Haikou, China

**Keywords:** C_3_–C_4_ intermediate photosynthesis, NADP-ME, cassava, C_4_ evolution, coexpression network

## Abstract

NADP-malic enzyme (NADP-ME) is a crucial enzyme in C_4_ photosynthesis, responsible for the decarboxylation of malate in bundle sheath cells, enhancing the photosynthetic efficiency of C_4_ plants. Cultivated cassava exhibits high photosynthetic efficiency and biomass, and previous studies classify it as a C_3_–C_4_ intermediate type. The biomass of cassava correlates positively with photosynthetic rate, and the promoter region of *MeNADP-ME3* contains insertion selected in cultivars different from wild ancestors. Four *MeNADP-ME* genes were identified in the cultivated cassava variety AM560, with promoter regions enriched in light-responsive elements. Phylogenetic and conserved domain analyses revealed that all subtypes are plastidic dicotyledonous types, closely related to *AtNADP-ME4*, with unique N-terminal domains in *MeNADP-ME2* and *MeNADP-ME3* specific to cassava, suggesting new functional roles. Subcellular localization showed predominant chloroplast localization, with greater involvement in leaf physiological processes in the cultivated variety SC205. These findings suggest that the NADP-ME family in cultivated cassava has been evolutionarily selected for photosynthesis. Further investigation revealed that *MeNADP-ME3* is highly expressed in leaves and regulated by light intensity. Co-expression network analysis of shade-treated transcriptomes and transcription factor-promoter predictions showed that Indel sites in the *MeNADP-ME3* promoter are bound by MeYABBY1, forming a regulatory network with other photosynthesis-related genes. This suggests that *MeNADP-ME3* plays a role in C_3_–C_4_ intermediate photosynthesis during the evolution from wild to cultivated cassava, with other family genes also evolving towards photosynthetic functions. Our study lays the foundation for future functional research on the *MeNADP-ME* family and provides insights into the mechanisms underlying the high photosynthetic efficiency of cultivated cassava.

## 1 Introduction

Cassava (*Manihot esculenta* Crantz), a dicotyledonous plant from the Euphorbiaceae family, is the third-largest source of carbohydrates in tropical regions, following rice and maize. It is an important staple for over 500 million people, known for its drought resistance and ability to grow in poor soils. Cultivated cassava is regarded as a crop with high tuberous root biomass, starch accumulation capabilities, and efficient photosynthetic performance (Moorthy and Padmaja, 2002; El-Sharkawy et al., 2012). Previous research has identified 98 varieties of wild cassava (*Manihot esculenta* ssp. *flabellifolia*), a small, climbing shrub primarily found in the regions bordering the Amazon rainforest and tropical savannas in Brazil and its surrounding areas (Luiz Joaquim Castelo Branco et al., 2017). This wild species is considered to be the direct ancestor of modern cultivated cassava. However, wild cassava exhibits significantly lower photosynthetic efficiency and starch accumulation compared to cultivated cassava, lacking the high light efficiency and high starch accumulation traits characteristic of the latter (Allem, 1999; An et al., 2016). Studies with ^14^C isotope feeding in cultivated cassava have revealed that 30%–50% of CO_2_ is converted into four-carbon acids, such as malate. However, due to the lack of Kranz anatomy, cultivated cassava is considered a C_3_–C_4_ intermediate type (Cock et al., 1987; El-Sharkawy, 2004). Phosphoenolpyruvate carboxylase (PEPC), a key enzyme in C_4_ and C_3_–C_4_ intermediate plants, is linked to photosynthetic carbon assimilation (Svensson et al., 2003). Analysis of 18 cassava cultivars showed a strong positive correlation between photosynthetic efficiency, tuberous root biomass, and MePEPC activity, further supporting the significant role of high MePEPC activity in enhancing cassava’s photosynthetic efficiency and tuberous root biomass (El-Sharkawy et al., 2008). These C_4_-related enzyme activities and C_3_–C_4_ intermediate traits significantly enhance the photosynthetic efficiency and tuberous root biomass of cultivated cassava.

The photosynthetic carbon assimilation process in C_4_ plants consists of two key components: carbon fixation and decarboxylation. Carbon fixation occurs in mesophyll cells, mainly facilitated by phosphoenolpyruvate carboxylase (PEPC), while decarboxylation takes place in bundle sheath cells. NADP-ME (NADP-malic enzyme, EC 1.1.1.40) plays a crucial role in C_4_ plants, catalyzing the conversion of malate to pyruvate and CO_2_ in bundle sheath cells, using NADP^+^ to increase CO_2_ concentration near Rubisco. This enhances Rubisco’s carboxylation activity while suppressing its oxygenation activity, thereby reducing photorespiration and improving carbon assimilation efficiency, a key factor in the high photosynthetic efficiency of C_4_ plants (Edwards and Andreo, 1992; Sage, 1999). C_4_-related enzymes already exist in C_3_ plants to confer resistance to stress conditions. In rice, the expression of NADP-ME increases under salt and drought stress, which assists in balancing levels of reactive oxygen species (ROS) and regulating malate concentrations in guard cells, thereby reducing stomatal aperture. The transfer of OsNADP-ME2 to *Arabidopsis* enhances its resistance to salt and osmotic stress (Chen et al., 2019). During the evolution towards C_4_, NADP-ME gradually shifted from its original C_3_ physiological roles, such as response to abiotic stress, to participating in the photosynthetic process (Tausta et al., 2002; Maier et al., 2011).

C_4_ enzymes were recruited into the photosynthetic process through mutations in the promoter regions, which altered the transcription factor–promoter regulatory networks. Additionally, the variation in copy number of C_4_ genes is a critical indicator of the evolutionary transition from C_3_ to C_4_ (Wang et al., 2009; Amy Lyu et al., 2023). Analysis of the cassava pan-genome reveals systematic Indel sites in the *MeNADP-ME3* gene between ancestral and cultivated varieties, along with an increased copy number of the *MeNADP-ME* gene family in cultivated cassava. Immunofluorescence localization shows that the MePEPC protein accumulates in the mesophyll cells around the vascular bundles in the cultivated cassava variety Arg7, a pattern not observed in the ancestral variety W14 (Xia et al., 2023). Single-cell transcriptome studies indicate that *MeNADP-ME* expression is higher in the vascular tissue of the leaves of cultivated cassava variety SC8 compared to mesophyll cells (Zang et al., 2023). Therefore, based on these previous experiments, we conclude that cultivated cassava exhibits C_3_–C_4_ intermediate photosynthesis, while wild cassava is classified as a C_3_ type. Despite the absence of a fully developed Kranz anatomy in cultivated cassava, the differential expression of *MePEPC* and *MeNADP-ME* between mesophyll and bundle sheath cells, combined with systematic indels in the *MeNADP-ME* gene, may have altered the regulatory network during the evolutionary transition from wild type to cultivar. This adaptation facilitates the recruitment of *MeNADP-ME* in photosynthesis. These findings suggest that the *MeNADP-ME* gene family could be a critical factor in the evolution from wild-type C_3_ cassava to a C_3_–C_4_ intermediate type in cultivated varieties. Previous research on the *NADP-ME* family has identified its critical role in C_4_ evolution, with extensive studies conducted on C_3_–C_4_ intermediate model species in *Flaveria* and C_4_ plants like maize. However, systematic investigations into its role in cassava’s C_3_–C_4_ intermediate photosynthesis are still lacking. Research into the light response and regulatory patterns of *MeNADP-ME* in cultivated cassava will enhance our understanding of C_4_ evolution in the Euphorbiaceae family and provide valuable genetic resources to improve the photosynthetic efficiency of C_3_ plants.

In this study, we investigated the evolutionary transition of the *MeNADP-ME* gene family from wild to cultivated cassava and its role in enhancing photosynthetic efficiency. Using the cultivated cassava genotype AM560 as a reference, we identified four *MeNADP-ME* genes and analyzed their promoter regions for cis-regulatory elements. We also examined their conserved domains and constructed a phylogenetic tree. Subcellular localization was performed by transiently transforming tobacco, revealing the evolutionary features of these genes. Using qRT-PCR, we analyzed the gene expression in various tissues of cultivated cassava SC205 and wild varieties W14 and FLA4047, with a particular focus on diurnal rhythm and abiotic stress responses in the cultivated variety. Finally, a bioinformatics-based co-expression network was constructed for shaded leaves of cultivated cassava, highlighting the photosynthetic role of the *MeNADP-ME3* gene family.

## 2 Materials and methods

### 2.1 Identification, chromosomal localization, and cis-regulatory element analysis of the *MeNADP-ME* gene family

We used BLASTP (Johnson et al., 2008) to compare the *Arabidopsis* NADP-ME protein sequences with the cassava reference genome (AM560-2-JGI-v8.1). Genes with an E-value ≤ 1e−5 were selected, and their chromosomal locations were determined. The chromosomal map was created using the “Gene Location Visualize from GTF/GFF” tool in TBtools (Chen et al., 2020). The conserved protein domains of the MeNADP-ME gene family were analyzed using the MEME suite (https://meme-suite.org/meme/), setting the motif number to 15. Protein analysis, including amino acid length, isoelectric point, molecular weight, and hydrophilic coefficient, was done using the ExPASy server (https://web.expasy.org/protparam). The subcellular localization of MeNADP-ME proteins was predicted using the Plant-mPLoc server (http://www.csbio.sjtu.edu.cn/cgi-bin/PlantmPLoc.cgi). To study cis-regulatory elements, the 3,000-bp upstream sequence of each gene was submitted to the PlantCARE database (https://bioinformatics.psb.ugent.be/webtools/plantcare/html/).

### 2.2 Plant materials and treatment methods

This study used cultivated cassava varieties KU50, TMS60444, SC205, and SC16 and wild varieties FLA4047 and W14. The cultivated varieties are widely planted and closely related to the reference genome AM560-2. TMS60444 is commonly used in tissue culture, while SC205 is frequently selected for research. KU50 exhibits superior resistance to abiotic stress compared to SC205, and SC16, developed by our team, shows a 40% higher yield than SC205. All plants were grown in Chengmai, Hainan, China (19.85°N, 110.08°E), with samples collected from storage roots, fibrous roots, leaves, and stems during the root expansion stage. Shading transcriptome analysis was performed using SC205 and SC16, chosen for their significant yield difference, hypothesizing divergent photosynthetic efficiencies. This approach helps eliminate variability in photosynthetic gene responses and construct a co-expression network to study *NADP-ME’s* role in cultivated cassava’s photosynthetic physiology. Shading treatment began at 8:00 a.m., with samples collected at 8:30 a.m. and 10:00 a.m., along with control samples. For circadian rhythm analysis, TMS60444 was sampled every 2 h from 6:00 a.m. to 8:00 p.m. Heat and shade-heat stress experiments were conducted with SC205 and KU50 to explore *MeNADP-MEs* responses and determine if they have physiologically diverged from the original C_3_ wild types. Control samples were collected at 10:00 a.m., followed by heat treatment at 43°C, with samples taken after 24 h and 48 h. Each treatment had three biological replicates, and all samples were immediately stored in liquid nitrogen.

### 2.3 RNA extraction and cDNA synthesis

Total RNA from all tissue samples was extracted using the RNAprep Pure Kit (TianGen Biotech., Ltd., Beijing, China; DP441) and treated to remove DNA, following the manufacturer’s instructions. RNA concentration and quality were measured with a NanoDrop, and RNA integrity was confirmed by 1% agarose gel electrophoresis. Only samples with intact RNA bands and a high quality (A260/280 ratio between 2.0 and 2.2) were used for reverse transcription. High-quality RNA was combined with the 5X Evo M-MLV RT Reaction Mix Ver.2 kit (Accurate Biology., Ltd., Wuhan, China; AG11728) to synthesize single-stranded cDNA following the manufacturer’s protocol. For RNA-seq, RNA integrity was further verified using the RNA Nano 6000 kit (Agilent Technologies, Santa Clara, CA, USA; 5067–1511) on the Bioanalyzer 2100 system.

### 2.4 Transcriptome cDNA library construction, sequencing, and raw data processing

First, mRNA was enriched using mRNA Capture Beads, followed by magnetic bead purification. The mRNA was then fragmented under high-temperature conditions. The fragmented mRNA served as the template for first-strand cDNA synthesis in the reverse transcriptase reaction system. End repair and addition of an A-tail were completed during the synthesis of the second cDNA strand. Adaptors were ligated, and target fragments were selected using Hieff NGS^®^ DNA Selection Beads (Yeasen Biotechnology Co., Ltd., Shanghai, China; 12601ES). The library was constructed by PCR amplification and sequenced on the Illumina Novaseq X Plus platform. For data processing, raw sequencing data were quality-controlled using fastp with default parameters (Chen et al., 2018). Bowtie 2 was used to align the clean reads to the ribosomal RNA database, removing reads mapped to ribosomal RNA. Unmapped reads were kept for transcriptome analysis (Langmead and Salzberg, 2012). Then, HISAT2 was used to align the paired-end reads to the cassava reference genome (AM560-2-JGI-v8.1) to generate read counts for each gene (Kim et al., 2019). Using the reference genome annotation file, the exon lengths of cassava genes were extracted, and FPKM values were calculated for each gene (Trapnell et al., 2010). The raw data for each sample were uploaded to the National Genomics Data Center, with quality control metrics and mapping rates provided in (**Supplementary Table S1**).

### 2.5 Quantitative real-time PCR analysis

For quantitative real-time PCR analysis, cDNA samples were diluted 20-fold. The expression levels of target genes were measured on a Bio-Rad CFX96 instrument using the 2X SYBR Green Pro Taq HS Premix II kit (Accurate Biology, Ltd., Wuhan, China; AG11702), with each sample analyzed in four technical replicates. The PCR cycling program began with a 30-s denaturation at 95°C, followed by 40 cycles of 95°C for 5 s and 60°C for 30 s. Melt curve analysis and cycle threshold (Ct) values were determined using CFX software (Bio-Rad). The cassava *Tubling* gene was used as the reference gene for relative expression analysis, as described in previous studies (Yao et al., 2014). Primers for *MeNADP-ME1*, *MeNADP-ME2*, *MeNADP-ME3*, and *MeNADP-ME4* were designed across exons from the CDS sequence and verified for specificity using Primer-BLAST (Ye et al., 2012). Primer sequences are provided in (**Supplementary Table S2**). Gene expression levels were calculated using the 2^−ΔΔCt^ method (Livak and Schmittgen, 2001).

### 2.6 Conserved protein domain and phylogenetic tree analysis

We downloaded the NADP-ME amino acid sequences from *Arabidopsis thaliana* (TAIR10) and cassava (AM560-2-JGI-v8.1) and analyzed conserved domains using the MEME suite (https://meme-suite.org/meme/), setting the motif number to 15. To construct the phylogenetic tree, BLASTP was used to identify homologous genes (E-value ≤ 1e−5) in *Arabidopsis thaliana* (TAIR10), rice (MSU-v7.0), maize (MaizeGDB-Zm-B73-REFERENCE-NAM-5.0), potato (MSU-v6.1), and sorghum (JGI-v2), and their protein sequences were retrieved. Homologous *NADP-ME* genes and sequences from *Flaveria robusta* (C_3_), *Flaveria floridana* (C_3_–C_4_), and *Flaveria bidentis* (C_4_) were based on a previous study (Taniguchi et al., 2021). The phylogenetic tree was constructed using the maximum likelihood (ML) method. Multiple sequence alignment was done using the Muscle tool in MEGA v. 11 (Tamura et al., 2021), followed by model selection with default parameters and 1,000 bootstrap replicates. The phylogenetic tree was visualized using iTOL (Letunic and Bork, 2024).

### 2.7 Subcellular localization analysis of cassava *MeNADP-MEs*

To construct the pG1300-MeNADP-MEs-GFP vector, we used the pCAMBIA1300-GFP vector. Primers were designed to amplify the CDS of each MeNADP-ME family member from the cDNA of mature leaves of the cassava cultivar SC205. CDS cloning was performed using 2X ApexHF CL PCR Master Mix (Accurate Biology, Ltd., Wuhan, China; AG12209). The pG1300-GFP vector was digested with KpnI-HF^®^ and SalI-HF^®^ (New England Biolabs). Both the cloned CDS and the digested vector were purified using 1% agarose gel electrophoresis. The ClonExpress Ultra One Step Cloning Kit V2 (Vazyme, Nanjing, China; C116) was used for homologous recombination, ligating the CDS fragments into the vector. The recombinant vectors were transformed into *Agrobacterium tumefaciens* strain GV3101. After reaching an OD600 = 0.9, the bacterial cells were resuspended in MES buffer (10 mM MgCl_2_, 10 mM MES, 100 µM AS) to OD600 = 9.0 and injected into the abaxial side of tobacco leaves. After 48 h in the dark, 1-cm^2^ sections of the transformed leaves were sampled for imaging on a FV3000 confocal microscope (Olympus, Japan), capturing bright field, chloroplast fluorescence (680 nm), and GFP fluorescence (488 nm) images, which were merged using FV31S-DT software (Olympus, Japan).

### 2.8 Weighted gene co-expression network analysis for shading transcriptome and transcription factor–promoter interaction prediction

Genes with the top 7,000 average FPKM values were selected and normalized using log2(FPKM+1). Sample clustering and soft threshold analysis were performed (**Supplementary Figure S1**). A soft threshold of 18 was chosen to construct the weighted gene co-expression network analysis (WGCNA) co-expression network using the R package version of WGCNA (Langfelder and Horvath, 2008). Photosynthesis-related gene sets were created by extracting cassava genes involved in light-harvesting antennae (KEGG pathway ID: map00196), photosynthetic electron transport (KEGG pathway ID: map00195), and carbon fixation (including the Calvin–Benson cycle and C_4_ genes; KEGG pathway ID: map00710) from the KEGG pathway database. The number of photosynthesis-related genes in each module was calculated, and the module with the highest number of photosynthetic genes was selected for further analysis. KEGG enrichment analysis was performed on all genes in this module (**Supplementary Figure S2**). Using the Plant TF Motifs Shift tool in TBtools, protein sequences in the module (AM560-2 JGI-v8.1) were analyzed with *Arabidopsis thaliana* as a reference to identify transcription factor binding motifs. The 3,000-bp upstream promoter regions of photosynthesis-related genes were extracted and scanned using the Fimo: Binding Motif Scan tool in TBtools to generate a predicted transcription factor–promoter interaction network. The network was visualized using Cytoscape 3.7.2 software.

### 2.9 Statistical analysis

Each qPCR sample was analyzed in four technical replicates. Tukey HSD significance analysis of the qPCR results was conducted using SPSS software (IBM Corp., Armonk, NY, USA; version 8.0). Differences were indicated by letter labeling, where different letters represent significant differences (p < 0.05). Figures were created using GraphPad Prism software (GraphPad Software, San Diego, CA, USA; version 8.0).

## 3 Result

### 3.1 The identification of *NADP-ME* family in cassava

**Figure 1:**
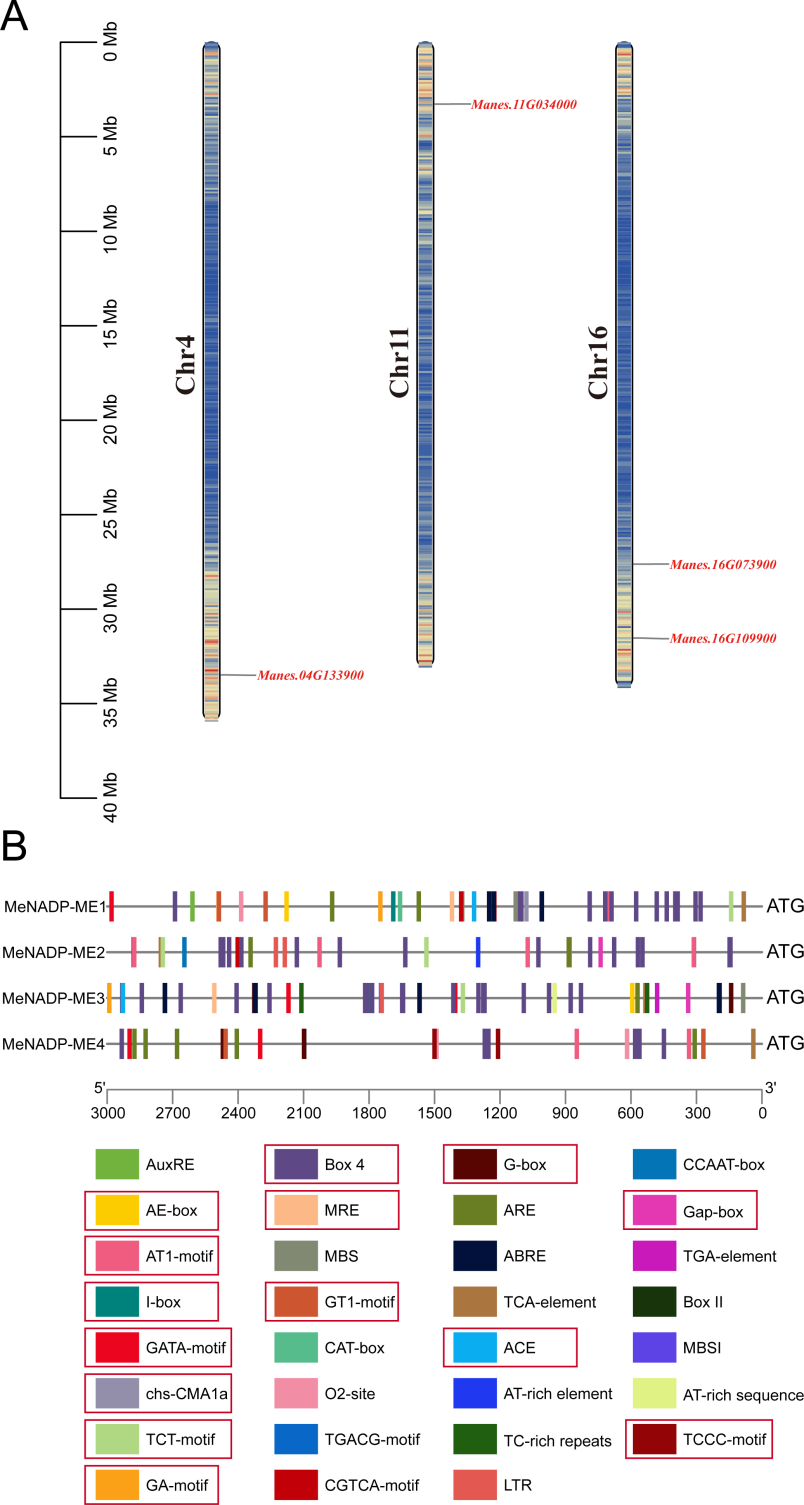
Chromosomal localization **(A)** and upstream cis-acting elements of the *NADP-ME* gene family in cassava **(B)**, the 5′ to 3′ direction represents the orientation of the promoter sequence, and the red box indicates the photosynthesis-related cis-regulatory elements.

A BLASTP search using *Arabidopsis* NADP-ME (TAIR10) as a query against the cultivated cassava AM560-2 dataset (Phytozome 13, E-value ≤ 1e−5) identified four distinct members of the *MeNADP-ME* family: *MeNADP-ME1*, *MeNADP-ME2*, *MeNADP-ME3*, and *MeNADP-ME4*, located on chromosomes 16, 16, 11, and 4, respectively (**Figure 1A**). Physicochemical analysis (**Table 1**) revealed that *MeNADP-ME1* has the fewest amino acids, lowest molecular weight, and smallest isoelectric point, while *MeNADP-ME2* exhibited the highest values for these properties. *MeNADP-ME3* and *MeNADP-ME4* had intermediate characteristics. Subcellular localization predictions indicated chloroplast localization for all four isoforms. Promoter analysis (**Figure 1B**, **Supplementary Table S3**) identified numerous light-responsive cis-regulatory elements, particularly in *MeNADP-ME1* and *MeNADP-ME3*, suggesting their involvement in photosynthetic regulation. Additionally, these genes contain hormone- and stress-responsive elements, although light-responsive elements dominate their regulatory mechanisms. These findings suggest that the *MeNADP-ME* family plays a role in the photosynthetic physiology of cultivated cassava.

**Table 1:** Basic physical and chemical properties of NADP-ME protein in cassava.

Gene name	Gene ID	Amino acid	Molecular weight	Isoelectric point	Hydrophilic coefficient	Predicted subcellular localization
*MeNADP-ME1*	Manes.16G109900	591	65,001.78	6.03	−0.100	Chloroplast
*MeNADP-ME2*	Manes.16G073900	661	73,450.93	8.38	−0.111	Chloroplast
*MeNADP-ME3*	Manes.11G034000	638	70,092.35	6.27	−0.134	Chloroplast
*MeNADP-ME4*	Manes.04G133900	643	70,771.27	6.73	−0.152	Chloroplast

### 3.2 Conserved domain and phylogenetic tree of the *MeNADP-MEs*

**Figure 2:**
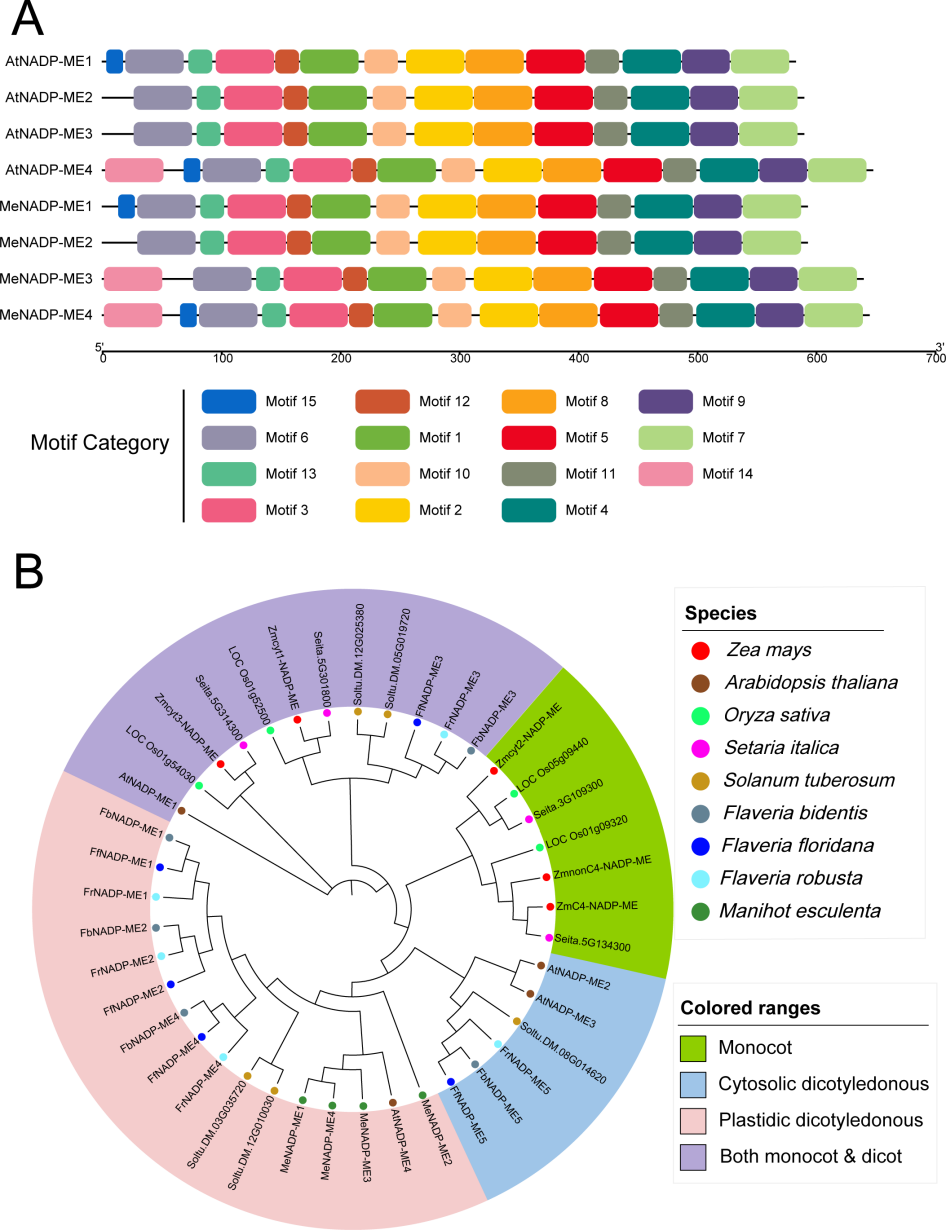
The conserved structure of *NADP-ME* was analyzed using NADP-ME protein sequences from *Arabidopsis* and cassava **(A)**; phylogenetic tree was constructed based on *NADP-ME* sequences from maize (C_4_), *Arabidopsis* (C_3_), *Flaveria robusta* (C_3_), *Flaveria floridana* (C_3_–C_4_), *Flaveria bidentis* (C_4_), potato (C_3_), rice (C_3_), sorghum (C_4_), and cassava (C_3_–C_4_) species, illustrating the evolutionary relationships and functional diversification of *NADP-ME* **(B)**.

We conducted a MEME analysis to investigate conserved motifs in the NADP-ME proteins of cassava and *Arabidopsis* (**Figure 2A**). The results showed that the primary structural differences between the two species’ NADP-ME proteins occur mainly in the N-terminal region. Specifically, *MeNADP-ME2*, *MeNADP-ME1*, and *MeNADP-ME4* shared high homology with similar *Arabidopsis* subtypes, while MeNADP-ME3 exhibited a unique N-terminal domain, lacking Motif15 compared to *MeNADP-ME*4 and *AtNADP-ME4*. We also constructed a phylogenetic tree with *NADP-ME* genes from species such as maize (C_4_), *Arabidopsis* (C_3_), *Flaveria* species, potato (C_3_), rice (C_3_), sorghum (C_4_), and cassava (C_3_–C_4_) (**Figure 2B**). The analysis revealed four distinct subgroups: monocot, both monocot and dicot, cytosolic dicotyledonous, and plastidic dicotyledonous types. The phylogenetic distances correlated with both evolutionary relationships and subcellular localization. All *MeNADP-ME* members were classified into the plastidic dicotyledonous subgroup, consistent with localization predictions. Notably, cassava *NADP-ME* genes were most closely related to *AtNADP-ME4*, providing insights into the functional adaptation of these genes across species.

### 3.3 Subcellullar localization and different expression of *MeNADP-MEs*

**Figure 3:**
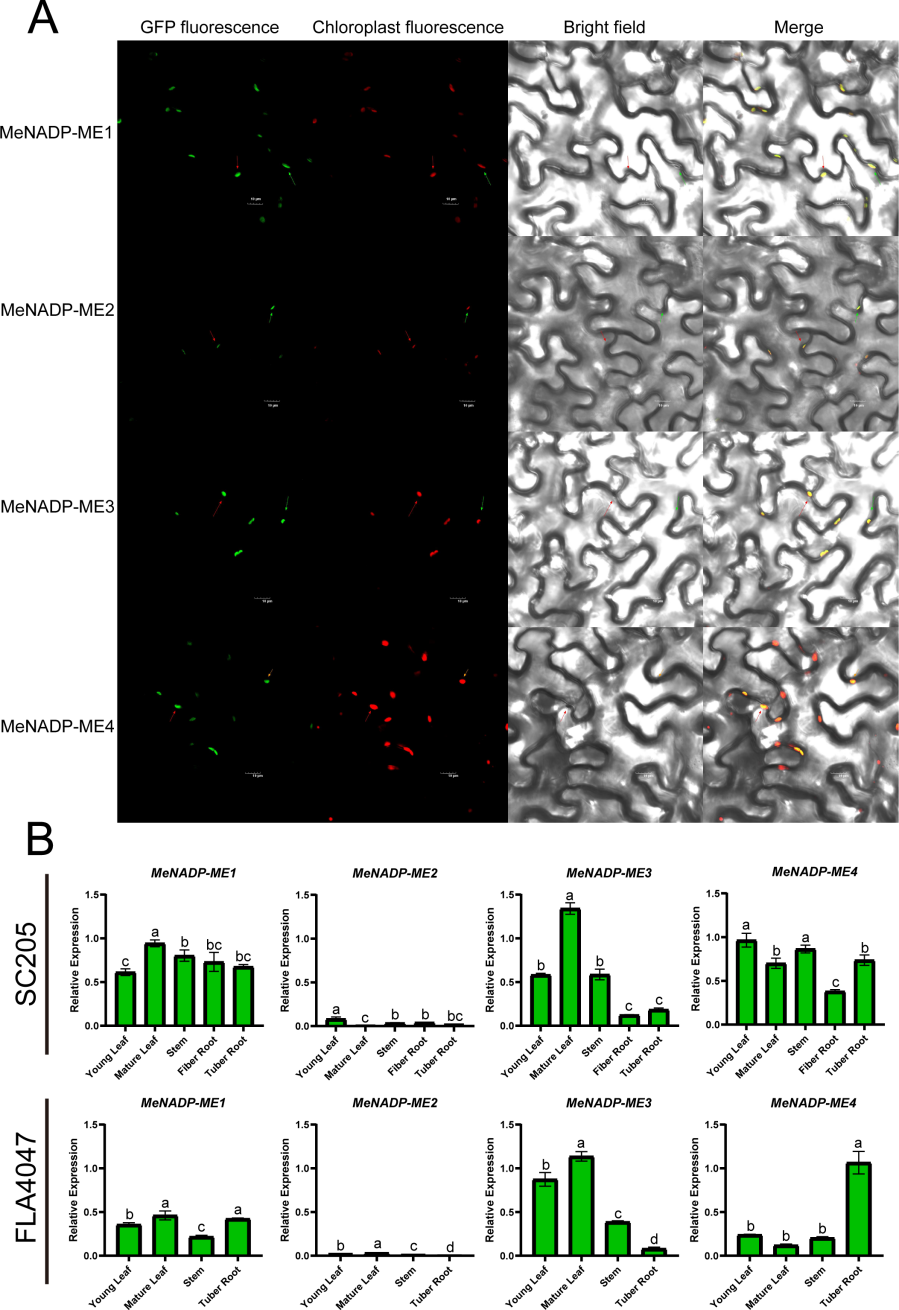
Subcellular localization of *MeNADP-ME* family (scale bar = 10μm) **(A)**; expression level changes of *MeNADP-ME* family in cassava cultivars SC205 and wild FLA4047 species **(B)**.

To investigate the subcellular localization of the *MeNADP-ME* gene family in cultivated cassava, we cloned the CDS regions of the four identified members from the AM560-2 reference genome, using TMS60444 as a template, and ligated them into a GFP vector driven by the CaMV 35S promoter. Transient expression in tobacco leaves (**Figure 3A**) revealed chloroplast localization for all *MeNADP-ME* members, consistent with phylogenetic and predictive analyses. Expression patterns in various tissues of cassava cultivar SC205 and wild species A4047 (**Figure 3B**) showed that in SC205, *MeNADP-ME1* was most expressed in mature leaves, *MeNADP-ME2* in young leaves, *MeNADP-ME3* in mature leaves, and *MeNADP-ME4* in young leaves and stems. In A4047, *MeNADP-ME1* was highly expressed in mature leaves and tuberous roots, while *MeNADP-ME2* and *MeNADP-ME3* were highest in mature leaves. *MeNADP-ME4* exhibited significantly higher expression in tuberous roots. These results highlight significant differences in the expression patterns of *MeNADP-ME* family members between cultivated and wild cassava, suggesting that the family has been selected to enhance leaf photosynthetic function in cultivated cassava, reflecting C_3_–C_4_ intermediate photosynthesis evolution.

### 3.4 Diurnal and stress conditional expression pattern of *MeNADP-MEs*

**Figure 4:**
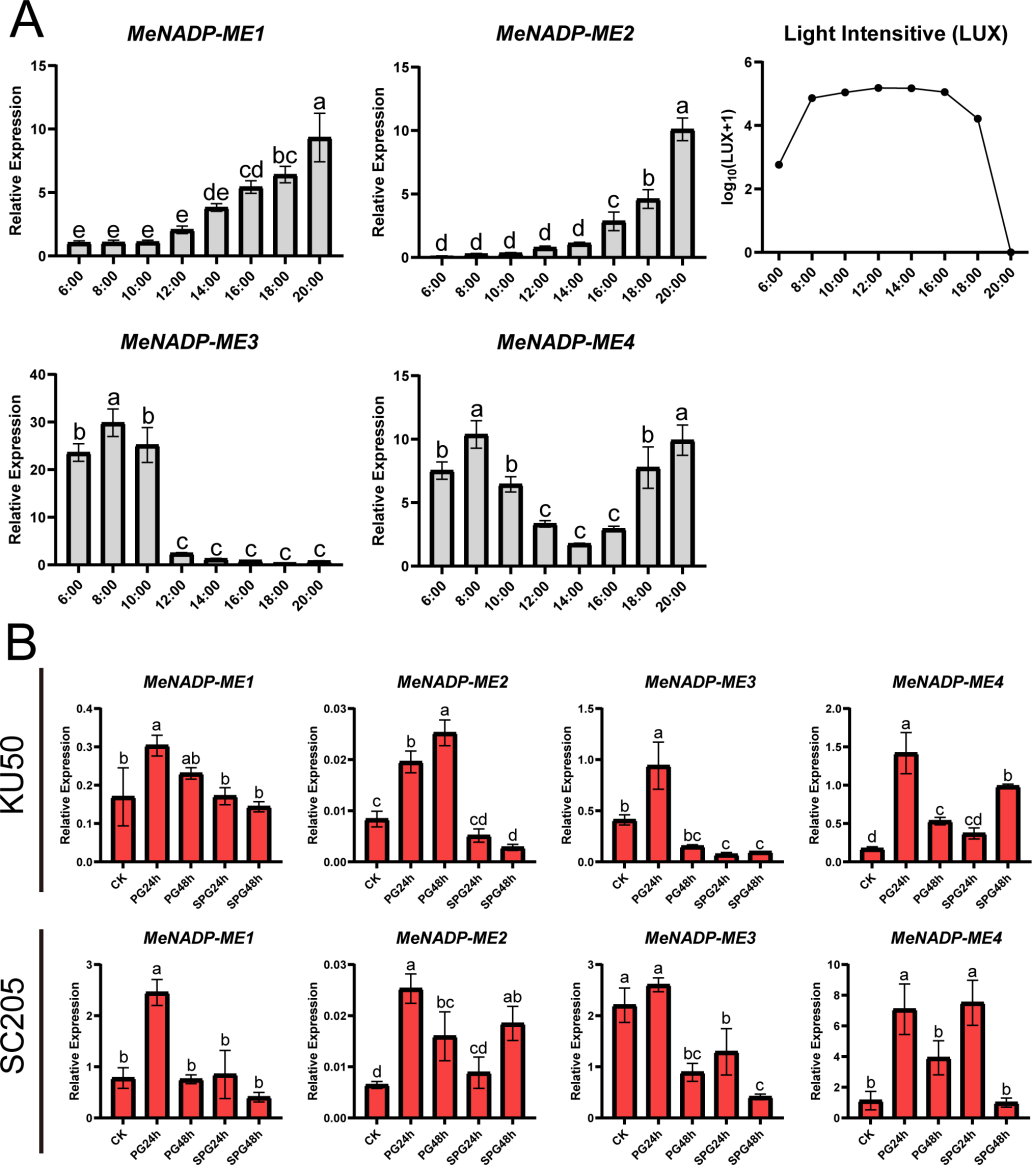
Diurnal expression of cassava cultivars TMS60444 *MeNADP-ME* (6:00–20:00) and light intensity changes (LUX) **(A)**; *MeNADP-ME* expression in cassava cultivars KU50 and SC205 under heat and heat-shading treatments **(B)**. **(B)** PG24h, heat treatment for 24 h; PG48h, heat treatment for 48 h; SPG24h, shade heat treatment for 24 h; SPG48h, shade heat treatment for 48 h. CK represents the control group, which corresponds to the untreated condition.

To investigate the diurnal response of the *MeNADP-ME* gene family in cultivated cassava and identify key members involved in photosynthetic activity, we analyzed their expression dynamics at different time points in the cultivar TMS60444 (**Figure 4A**). The expression patterns of *MeNADP-ME1* and *MeNADP-ME2* were similar, showing low levels in the morning, increasing after noon, and peaking at night. In contrast, *MeNADP-ME3* and *MeNADP-ME4* exhibited a strong positive correlation with light intensity, particularly between 6:00 and 8:00 a.m., when light intensity sharply increased, leading to a peak in expression. After 10:00 a.m., expression gradually decreased, with *MeNADP-ME4* showing a second peak at 20:00. Given the involvement of *NADP-ME* genes in C_3_ species’ response to abiotic stress, we further examined the family’s response to heat and combined heat and shading stress in cassava cultivars KU50 and SC205 (**Figure 4B**). In KU50, gene expression was generally upregulated at PG24h, with *MeNADP-ME2* significantly upregulated at PG48h, while other genes were downregulated. Under shade-heat stress, all genes, except *MeNADP-ME4*, were downregulated. In SC205, *MeNADP-ME1*, *MeNADP-ME2*, and *MeNADP-ME3* peaked at PG24h, significantly decreasing by PG48h. Under shade-heat stress, *MeNADP-ME2* was upregulated at SPG24h. In conclusion, *MeNADP-ME3* and *MeNADP-ME4* were identified as key contributors to photosynthesis in response to increased light intensity. Heat stress induced upregulation of *MeNADP-ME* genes, peaking at 24 h, while no significant changes occurred at 48 h or under combined heat and shading stress. These findings suggest that the *MeNADP-ME* gene family in cultivated cassava reflects an evolutionary trend toward C_4_ photosynthesis while retaining characteristics of its C_3_ ancestral species, underscoring the unique physiological features of its intermediate C_3_–C_4_ *MeNADP-ME* genes.

### 3.5 Construction of the photosynthetic gene co-expression network reveals new regulatory factors in cassava

**Figure 5:**
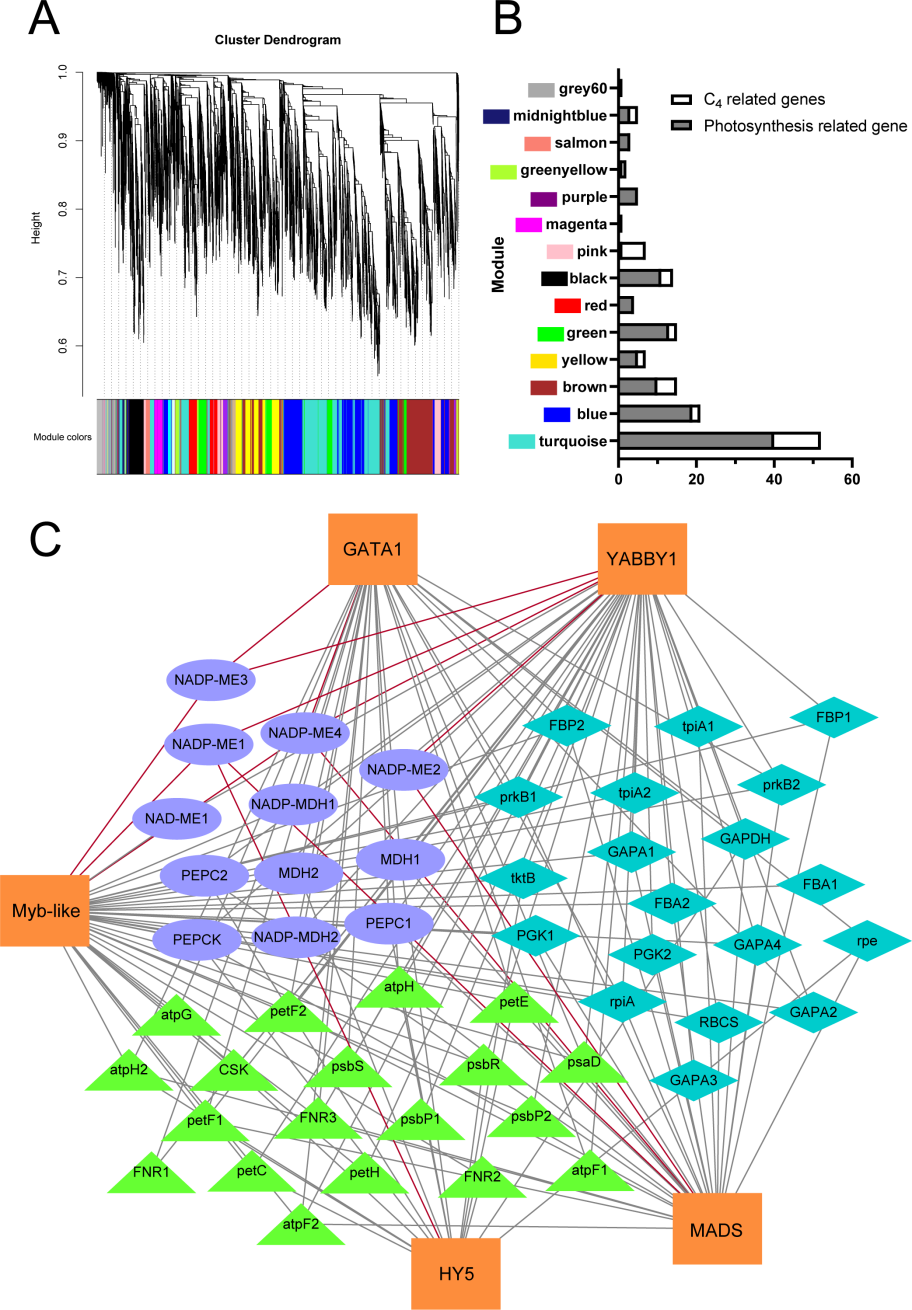
Co-expression network of transcriptomes under shading treatment in cassava cultivars SC205 and SC16 **(A)**; number of photosynthesis-related genes in each module **(B)**; transcription factor–gene interaction network of the Turquoise module **(C)**, where ellipse shapes represent C_4_ genes in cassava, diamond shapes represent genes related to the Calvin–Benson cycle, and triangle shapes represent genes related to the photosynthetic electron transport chain. The red lines represent the interaction between the promoter regions of the *MeNADP-ME* family and transcription factors.

**Figure 6:**
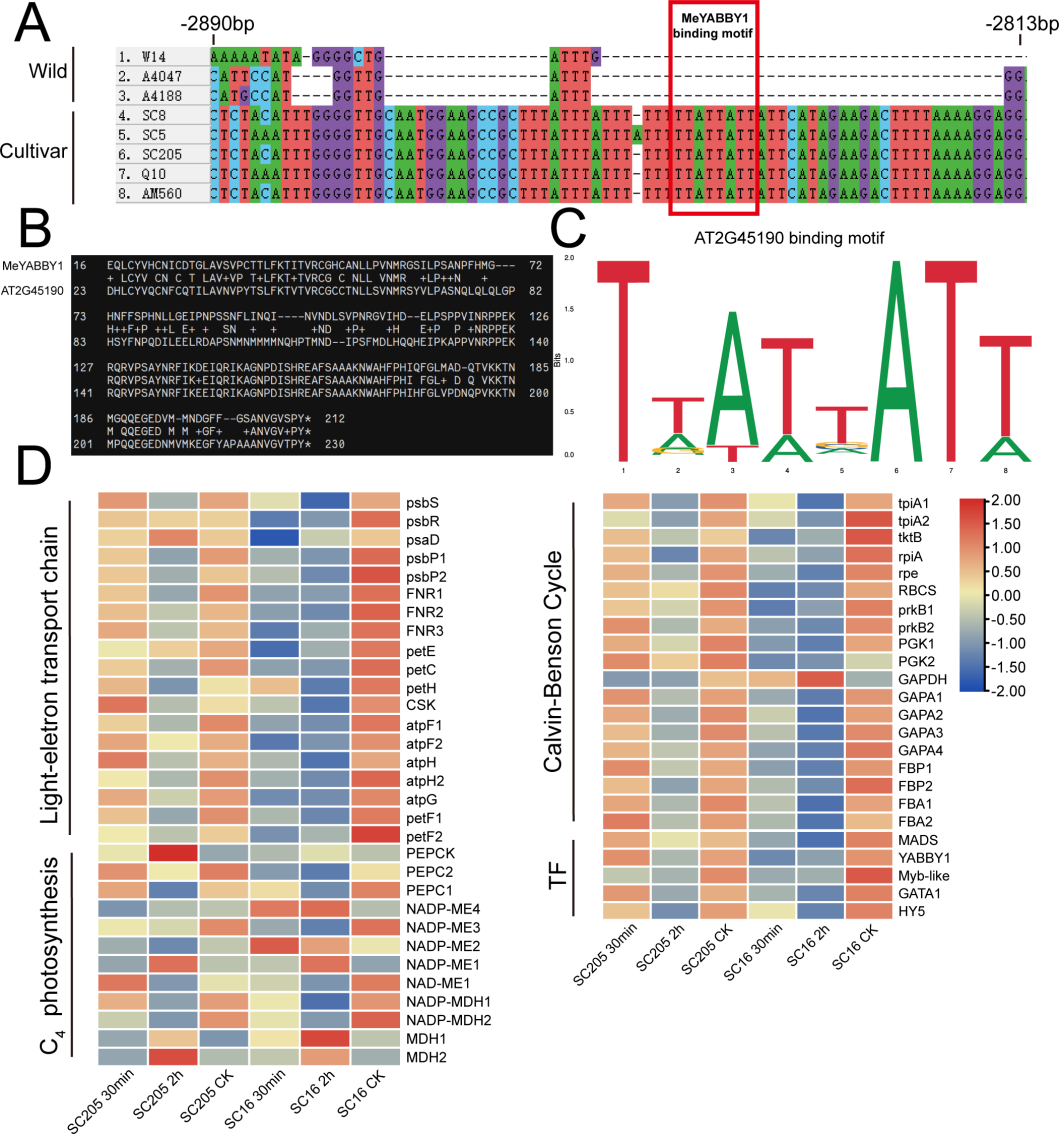
Predicted binding sites of MeYABBY1 on the Indel region, with the red rectangle indicating the MeYABBY1 binding motif **(A)**; blast alignment of MeYABBY1 with the most homologous transcription factor in *Arabidopsis*, AtYABBY1 (*AT2G45190*) **(B)**; the promoter motif sequences interacting with AtYABBY1 in the Jaspar transcription factor database **(C)**; heatmap of expression levels of photosynthetic electron transport chain, C_4_, Calvin–Benson cycle, and predicted interacting transcription factors (TF) in the Turquoise module under shading treatment **(D)**. CK represents the control group, which corresponds to the untreated condition.

We conducted co-expression network analysis of the transcriptome under shading treatment in the cultivated cassava varieties SC16 and SC205, identifying 18 modules with distinct expression patterns. Notably, the Turquoise module, which included all members of the *MeNADP-ME* gene family, was enriched with photosynthesis-related and C_4_ photosynthesis genes (**Figures 5A, B**). KEGG analysis revealed significant enrichment in the “carbon fixation in photosynthetic organisms” pathway (**Supplementary Figure S2**). Under shading conditions, cassava C_4_ genes, photosynthetic electron transport chain genes, and photosynthetic carbon assimilation genes, including the *MeNADP-ME* family, were regulated by transcription factors such as GATA1 (*Manes.03G154500*), YABBY1 (*Manes.02G035700*), Myb-like (*Manes.15G163100*), HY5 (*Manes.12G040300*), and MADS (*Manes.10G099000*) (**Figure 5C**). Heatmap analysis further showed that *MeNADP-ME3* exhibited an expression pattern consistent with most photosynthesis-related genes and their transcriptional regulators (**Figure 6D**), while *MeNADP-ME1*, *MeNADP-ME2*, and *MeNADP-ME4* exhibited opposite trends in SC16. This highlights *MeNADP-ME3* as a key gene in the intermediate C_3_–C_4_ photosynthetic physiology of cultivated cassava. Transcription factor-promoter prediction suggested that MeYABBY1 regulates *MeNADP-ME3* and binds to its systemic promoter Indel site, potentially playing a critical role in recruiting *MeNADP-ME3* into the C_3_–C_4_ photosynthetic regulatory network (**Figures 6A–C**).

## 4 Discussion

*NADP-ME* is widely distributed in both monocot and dicot plants. Previous research has categorized it into four subgroups: monocot, both monocot and dicot, cytosolic dicotyledonous, and plastidic dicotyledonous types (Wheeler et al., 2005; Alvarez et al., 2013). The Asteraceae family, belonging to dicots, includes species from the genus *Flaveria*, which encompasses C_3_, C_3_–C_4_ intermediate, C_4_-like, and C_4_ species. These species are considered an important model for studying the evolution of C_4_ photosynthesis, highlighting the dynamic process of C_4_ evolution (Adachi et al., 2022). To explore whether MeNADP-MEs protein shares similarities with the C_3_–C_4_ intermediate type of *Flaveria*, we included *NADP-ME* family members from *Flaveria* species representing C_3_, C_3_–C_4_, and C_4_ types in our phylogenetic analysis. The results indicated that the evolutionary relationships of *NADP-ME* are more closely aligned with species phylogeny and protein subcellular localization. According to the APG IV classification (Group et al., 2016), the Euphorbiaceae family, to which cassava belongs, is most closely related to the Brassicaceae family, while it is more distantly related to the Solanaceae and Asteraceae families. This evolutionary distance is reflected in the closer phylogenetic relationship between the cassava and *Arabidopsis NADP-ME* families, while the cassava *NADP-ME* family is more distantly related to members of *Flaveria* in the Asteraceae family and to those in the Solanaceae family, such as potato. The monocots, including rice, maize, and sorghum, show significant differences from the cassava *NADP-ME* family (**Figure 2B**). *MeNADP-ME* in cassava is most closely related to *AtNADP-ME4* in *Arabidopsis*, suggesting that they may share similar physiological functions in the chloroplast. Notably, in the conserved domain analysis, *MeNADP-ME2* lacks two conserved domains compared to the other cassava members and is more distantly related to *AtNADP-ME2* and *AtNADP-ME3* (**Figure 2A**). In contrast, *MeNADP-ME3* lacks Motif14, and no single *Arabidopsis* protein contains only Motif15 while lacking Motif14. This suggests that these two genes are unique to cassava. These structural differences are linked to the expansion of gene families during cassava’s evolutionary development towards C_4_ photosynthesis.

Previous studies suggest that *NADP-ME* in C_4_ plants evolved from the chloroplast-localized *NADP-ME* in their C_3_ ancestors, with these C_3_ chloroplast *NADP-MEs* originated from cytosolic *NADP-MEs* that did not participate in photosynthesis (Tausta et al., 2002; Maier et al., 2011). Furthermore, it has been proposed that as more *NADP-ME* members localize to the chloroplast, the evolutionary trend towards C_4_ photosynthesis becomes more pronounced. In particular, in C_4_ plants, *NADP-ME* plays a crucial role in bundle sheath cells by facilitating the decarboxylation reaction. This adaptation likely originated from the chloroplast-localized *NADP-ME* in C_3_ plants, which was subsequently selected and optimized for the chloroplast environment of C_4_ photosynthesis. As C_4_ photosynthesis evolved, the elevated expression of *NADP-ME* in the chloroplast became a hallmark of its role in driving C_4_ physiological functions (Langdale, 2011; Rao and Dixon, 2016; Shi et al., 2020). Phylogenetic analysis reveals that maize contains five *NADP-ME* members, two of which are localized to the chloroplasts. In contrast, all *NADP-ME* members in cassava are chloroplast-localized, with no cytosolic *NADP-ME* present, a feature unique to cassava (**Figure 3A**). This suggests that cassava has evolved a greater number of *NADP-ME* subtypes localized to the chloroplast, involved in physiological processes within the chloroplast. Additionally, a comparison of gene expression in cultivated and wild cassava species across different tissues (**Figure 3B**) shows that, compared to the wild-type A4047, the cultivated variety SC205 exhibits a greater involvement of *MeNADP-ME* in leaf physiological processes, while the wild type shows a more physiologically active subtype in underground tissues. These spatial expression patterns, observed in both subcellular and tissue-specific locations, provide strong evidence for the evolutionary shift of cultivated cassava from a C_3_ wild type to a C_3_–C_4_ intermediate type.

We also investigated the response of the *MeNADP-ME* gene family in cultivated cassava under diurnal rhythms and abiotic stress conditions (**Figures 4A, B**). Diurnal rhythm analysis indicated that *MeNADP-ME1* and *MeNADP-ME2* share similar physiological functions, predominantly operating during the night. In contrast, *MeNADP-ME3* and *MeNADP-ME4* are key genes involved in the response to changes in light intensity. Additionally, *MeNADP-ME4* exhibits high expression at night. We hypothesize that the ancestor of *MeNADP-ME* was a gene expressed during the night, and through selective pressure, it gradually began to function during the photosynthetic period. *MeNADP-ME4* shows clear signs of this selective process. Promoter cis-element analysis revealed a high proportion of light-responsive regulatory elements, and several stress-responsive elements, in these four genes (**Figure 1B**). Although both *MeNADP-ME1* and *MeNADP-ME3* contain over 20 light-responsive elements, the transcriptional levels of *MeNADP-ME1* do not fully align with the light cycle, suggesting that it is primarily regulated by factors beyond light response. In contrast, *MeNADP-ME3* is tightly correlated with light intensity, indicating that it functions as a core gene in the C_3_–C_4_ intermediate photosynthetic decarboxylation process in cassava. Our study of the expression patterns of the *MeNADP-ME* gene family in cultivated cassava under abiotic stress conditions reveals that it retains the physiological functions of its C_3_ wild relatives, consistent with the role of *NADP-ME* in stress responses in C_3_ plants. Previous research has shown that *NADP-ME* plays a critical role in stress responses by regulating cellular osmotic potential (Chen et al., 2019). Combined with the subcellular localization results, NADP-ME in cultivated cassava is likely involved in physiological processes that maintain chloroplast stability, particularly under abiotic stress conditions. Early studies have shown that during the evolution of C_4_ species, C_4_ genes were recruited into the photosynthetic process through structural variations in the promoter regions, facilitated by transcription factor–gene networks (Monson, 1999, 2003; Schlüter and Weber, 2020). The promoter region of *MeNADP-ME3* exhibits such structural variation, with an insertion mutation occurring during the evolutionary process from the wild type to the cultivated species, altering the transcriptional regulation pattern. First, its expression is significantly higher in the cultivated species’ leaves compared to other tissues, a feature not present in the wild species. Second, its expression increases only in response to increased light intensity, a feature not shared by other subtypes. Finally, it does not show a sustained response under prolonged heat stress. These characteristics suggest that *MeNADP-ME3* is gradually diverging from the physiological functions of its homologous C_3_ subtype, transitioning towards a role in photosynthesis.

In the constructed co-expression network of the cassava shading transcriptome (**Figures 5A, B**), *MeNADP-ME3* further exhibits distinct features. It is not only closely correlated with the expression trends of other photosynthetic genes within the module but also aligns with changes in transcription factor expression patterns (**Figure 6D**). Through the prediction of the transcription factor–promoter interaction network, we found that MeYABBY1 interacts with the systematic indel sites of *MeNADP-ME3* (**Figures 5C**, **6A–C**). Although members of the YABBY transcription factor family are typically associated with leaf development (She et al., 2022; Shen et al., 2022; Shi et al., 2024), some studies suggest that overexpressing *IaYABBY2* can enhance photosynthetic capacity in *Incarvillea arguta* (Sun et al., 2014), supporting the potential role of *YABBY* family members in the regulation of photosynthesis. This suggests that MeYABBY1 may play a role in the regulation of photosynthesis in cultivated cassava. We predict that MeYABBY1 interacts with multiple photosynthesis-related genes and other genes involved in C_4_ photosynthetic processes, such as *MePEPC1*, indicating that it is one of the core regulators in the C_3_–C_4_ transitional regulatory network of cultivated cassava. The co-regulation and similar expression patterns of *MeNADP-ME3* with *MePEPC1* and other photosynthesis-related genes further suggest that *MeNADP-ME3*, together with *MePEPC1*, contributes to the physiological process of C_4_ carbon fixation in cultivated cassava. In the shaded transcriptome, other *MeNADP-ME* subtypes are more involved in the physiological processes following shading.

Overall, this study found that during the evolutionary process from wild to cultivated varieties, the promoter region of *MeNADP-ME3* in cassava underwent selection, being recruited by transcription factors such as MeYABBY1, which altered its expression pattern and formed a transcription factor–gene regulatory network with other photosynthetic genes, thus participating in photosynthesis. Another interesting finding is that all *NADP-ME* gene members in cultivated cassava are localized in the chloroplasts, which is rare in other species. However, other *MeNADP-ME* subtypes in cultivated cassava have not yet evolved to respond to light and participate in photosynthesis, but subcellular localization indicates that they are located in the chloroplasts, showing a trend towards being recruited into photosynthesis. Given that cassava is a tropical plant, its adaptation to tropical environments may significantly influence its domestication process.

## Data Availability

All the raw transcriptome sequencing data generated from the leaf shading treatment during this study have been deposited at the National Genomics Data Center (https://ngdc.cncb.ac.cn/) as a BioProject under accession number PRJCA032135 (Shade treatment of cultivar Cassava SC16, SC205). The sequencing reads are available in the GSA database under the same BioProject number.
