# Supplementary material for: Identification of the cassava NADP-ME gene family and its response and regulation in photosynthesis
Source: Front Plant Sci. 2025 Feb 27;16:1525193. doi: 10.3389/fpls.2025.1525193 (PMC11903705; doi:10.3389/fpls.2025.1525193)
Supplement: Supplementary file 1 [file DataSheet1.docx]

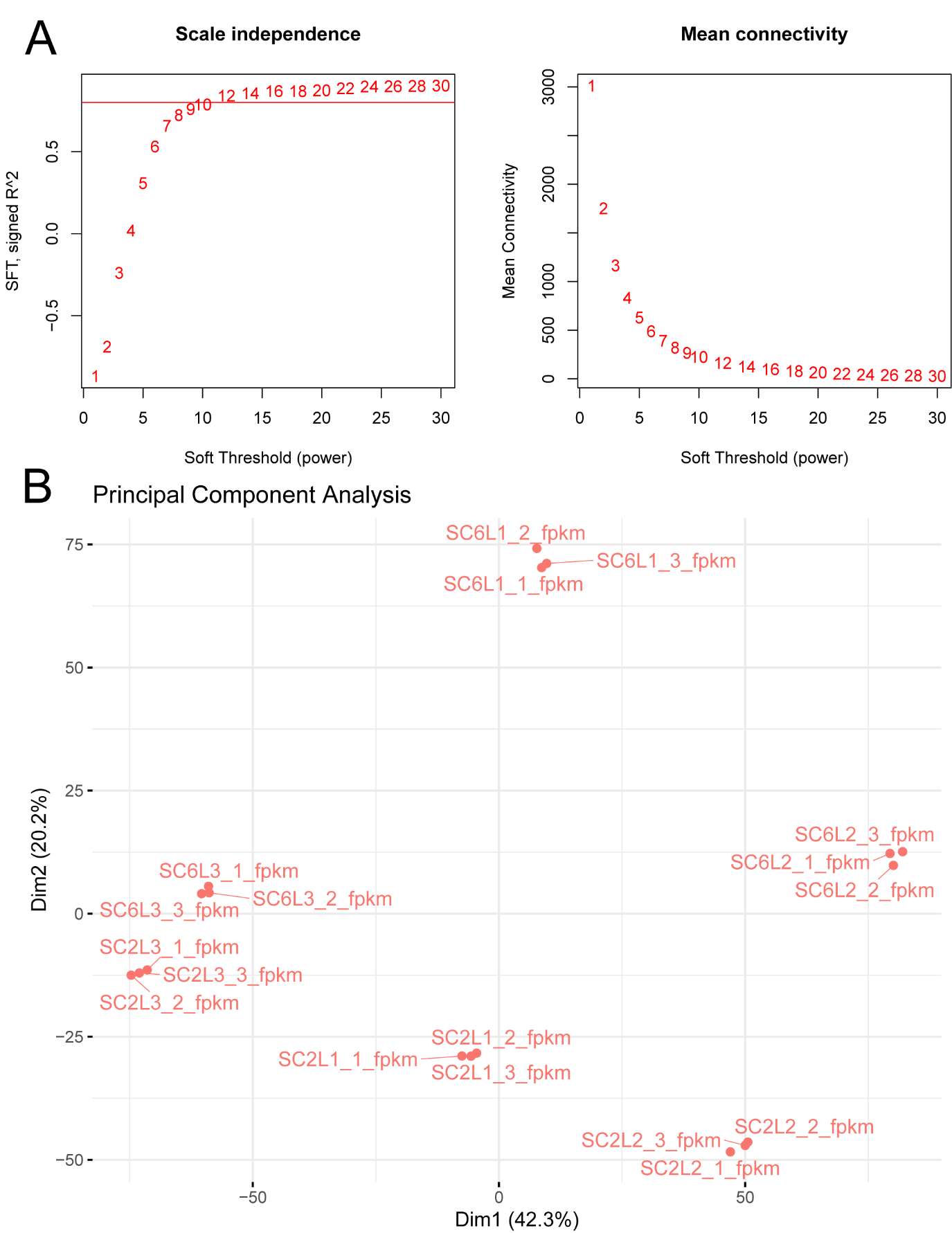


**Figure S1**. Soft-threshold Power Selection for Scale-free Topology and Mean Connectivity (A) and Principal Component Analysis (PCA) of Sample Clustering (B) in WGCNA

**
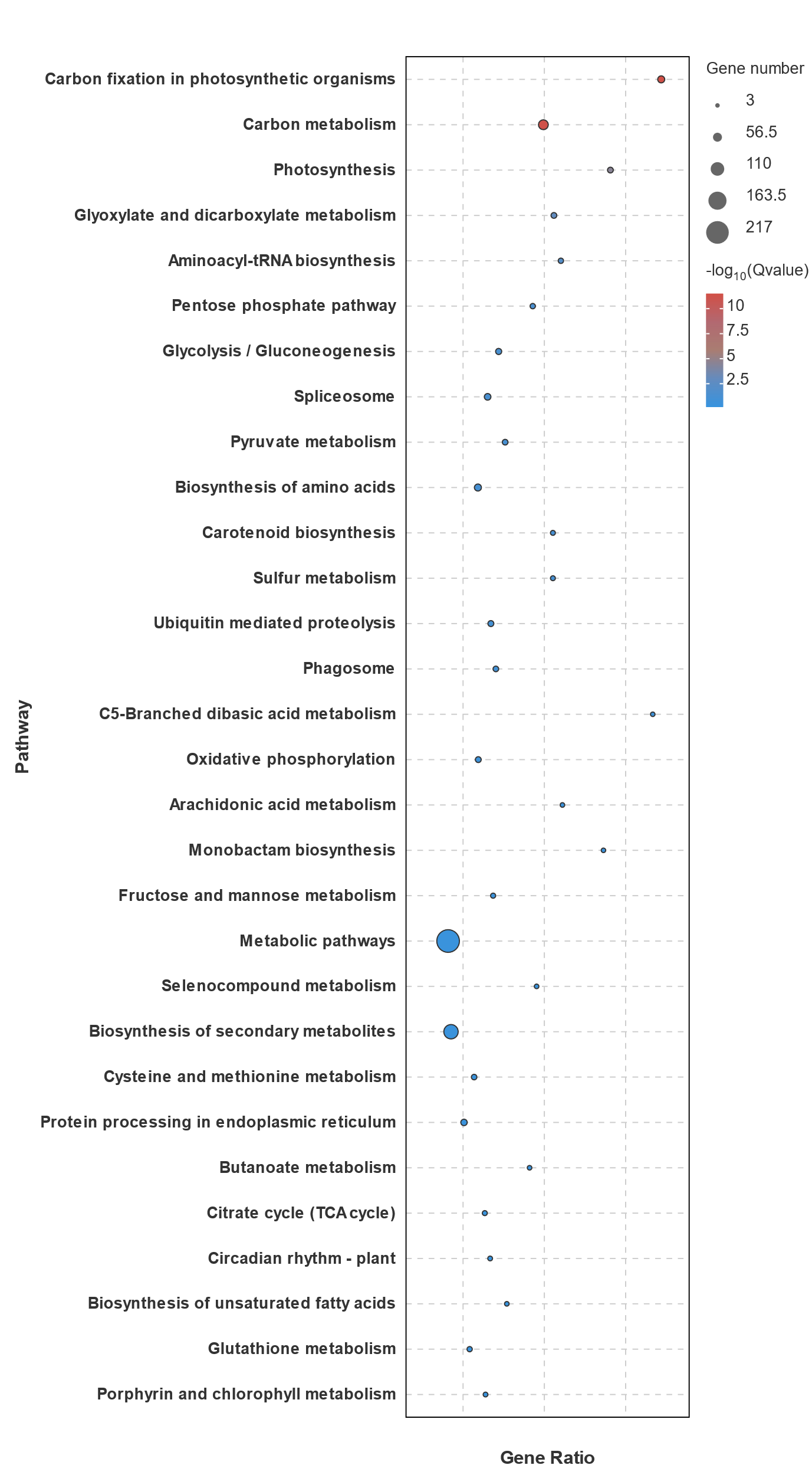
**

**Figure S2.** KEGG pathway enrichment analysis of the Turquoise module. Pathways enriched in the Turquoise module are listed on the left, with dot size representing the number of genes involved in each pathway and color indicating the -log₁₀(Q-value) for significance. Larger dots indicate pathways with more genes, while darker red represents higher significance.
